# 

**Published:** 1904-12

**Authors:** 


### INDEX TO VOLUME XXV.

A

Academy of Stomatology, 51, 137, 297, 443, 729, 783, 866
Advantages of co-operation in our profession, 43
Ainsworth, George C., D.D.S., Some thoughts regarding methods, and a new appliance for moving dislocated teeth into position, 481
Allen, Freeman, M.D., Recent methods in the administration of anæsthetics, 665
American Dental Society of Europe, 229
American Medical Association, Section on Stomatology, 200, 384, 398, 624, 772
American Society of Orthodontists, 562
An apology to Dr. L. C. Bryan, 153
Andrews, R. R., A.M., D.D.S., The vital action of the dental pulp, 759
Anema, R., D.D.S., The interest of the dentist *versus* the interest of the public, 706
An instructive case of hypertrophied pulp, 702
Ankylosis of the jaws, 764
Antiseptic science in Chicago, 543
Apparent trifles: their importance in dental practice, 283
A reply to Dr. Rhein's answer, 505.
Arkansas State Board of Dental Examiners, 824
Arrington, Dr. B. F., Pyorrhœa alveolaris, 274
Art of carving and baking porcelain teeth and crowns, 1

B

Baker, Dr. H. A., Treatment of protruding and receding jaws by the use of the intermaxillary elastics, 344
Baker, Lawrence W., D.M.D., A report on orthodontia, 843
Baldwin, A. E., M.D., D.D.S., LL.B., Some phases of dental education, 696
Banquet, Fourth International Dental Congress, 561, 656
Banquet to Dr. C. T. Stockwell, 953
Banquet to Dr. Scholl, 566
Bibliography:
A Compend of Dental Pathology and Dental Medicine. By Geo. W. Warren, A.M., D.D.S., 307
A Group of Distinguished Physicians and Surgeons of Chicago. By F. M. Sperry, 637
An Epitome of Inorganic Chemistry and Physics. By A. McGlannan, M.D., 69
A Text-Book of Dental Pathology and Therapeutics for Students and Practitioners. By Henry H. Burchard, M.D., D.D.S., 890

BIBLIOGRAPHY:

Essentials of Materia Medica, Therapeutics, and Prescription Writing. By Henry Morris, M.D., 896

Essig and Koenig's Dental Metallurgy. By Charles J. Essig, M.D., D.D.S., 950

General Catalogue of Medical Books, 953

Hale's Epitome of Anatomy. By Henry E. Hale, A.M., M.D., 152

Index der deutschen zahnärztlichen Literatur und zahnärztliche Bibliographie. By Prof. Dr. Port, 549

Irregularities of the Teeth and their Correction. By J. N. Farrar, M.D., D.D.S., 454

Irregularities of the Teeth and their Treatment. By Eugene S. Talbot, M.D., D.D.S., M.D., LL.D., 452

Manual of Materia Medica and Pharmacy. By E. Stanton Muir, Ph.G., V.M.D., 735

Normal Histology. By Edward K. Dunham, Ph.B., M.D., 951

Organic and Physiologic Chemistry. By Alexius McGlannan, M.D., 308

Sunshine Thoughts for Gloomy Hours. By George H. Chance, D.D.S., M.D., 454

The History of a History, 952

Bill regulating dental practice in the District of Columbia, 228

Bills, the collection of, 523

BIOGRAPHICAL SKETCH:

J. Foster Flagg, D.D.S. By William H. Trueman, D.D.S., 72

Boutwell, Leslie Barnes, D.M.D., The collection of bills, 523

C

California Board of Dental Examiners, 962

California State Dental Association, 400

Canadian Dental Association, 568

Card system for keeping dental records and accounts, 19

Caush, Douglas E., L.D.S., The development of hard tissue in the pulp of human teeth, 240

Central Dental Association of Northern New Jersey, 319

Chaddock, Charles Gilbert, M.D., Hypnotism, 686

Chittenden, Charles C., D.D.S., The evolution of standards in dental education, 698

Colorado State Board of Dental Examiners, 480

Correction, 735

Correction from Harvard, 225

Correction notice, 451

Correction of article in March number, 314

Correction of a serious error, 151

Correction of Stenographer's reports, 312

Crandall, G. C., B.S., M.D., Report of a case of Vincent's angina and stomatitis, 851

Cryer, M. H., M.D., D.D.S., Impacted teeth: their diagnosis, liberation, and extraction, 321
Cumston, Charles Greene, M.D., Dentigerous cysts, 256
CURRENT NEWS:
American Dental Society of Europe, 229
American Society of Orthodontists, 562
Arkansas State Board of Dental Examiners, 824
Banquet to Dr. C. T. Stockwell, 953
Banquet to Dr. Scholl, 566
Bill regulating dental practice in the District of Columbia, 228
California Board of Dental Examiners, 962
California State Dental Association, 400
Canadian Dental Association, 568
Central Dental Association of Northern New Jersey, 319
Colorado State Board of Dental Examiners, 480
Dental Board of New South Wales, 230
Dental Commissioners of Connecticut, 399, 479, 560
"F. D. I." Fédération Dentaire Internationale, 562, 661
First District Dental Society of New York, 320, 958
Florida State Dental Society, 87
Form of agreement of the State Board of New Jersey, 86
Fourth International Dental Congress, 457, 462, 471, 554, 561, 642, 648, 656, 657, 659, 820
Fraternal Dental Society of St. Louis, 230
Georgia State Dental Society, 480
Hartford Dental Society, 958
Harvard Dental Alumni Association, 744
Illinois State Dental Society, 319
Institute of Dental Pedagogics, 957
International Dental Congress—report of committee on prize essays, 820
Interstate Dental Fraternity of the United States and Canada, 88, 663
Iowa State Dental Society, 230
Kentucky State Dental Association, 318
Lebanon Valley Dental Association, 567
Minnesota State Board of Dental Examiners, 229, 400
Minnesota State Dental Association, 228
Mississippi Valley Medical Association, 743
Montana State Dental Society, 318
National Association of Dental Examiners, 229, 461, 561, 660, 821
National Association of Dental Faculties, 397, 563
New Haven Dental Association of Connecticut, 231
New Jersey State Board of Registration and Examination in Dentistry, 318, 823
New Jersey State Dental Society, 229, 470
Northern Indiana Dental Society, 399, 744, 823
Northern Iowa Dental Society, 568

CURRENT NEWS:
Northern Ohio Dental Association, 480
Ohio State Dental Society—thirty-ninth annual meeting, 959
Pennsylvania Association of Dental Surgeons, 232
Pennsylvania State Board of Dental Examiners, 397
Pennsylvania State Dental Society, 456
Programme of the American Medical Association, Section on Stomatology, 398
Programme of the F. D. I., 663
Reading Dental Society, 231
Resolutions American Dental Trade Association, 662
South Dakota State Board of Dental Examiners, 397, 904
Southern Branch, National Dental Association, 160, 904
Southern Dental Society of New Jersey, 231
Southwestern Iowa Dental Society, 744
Supreme Chapter, Delta Sigma Delta, 568, 662
The Interstate Dental Fraternity of the United States and Canada, 88, 663
The New York Institute of Stomatology, 159
Universal Exposition—hotel rates, 657
Wisconsin State Dental Society, 568
Curtis, G. Lenox, M. D., Ankylosis of the jaws, 764

D

Dawbarn, R. H. M., M.D., Malignant growths in the mouth, 911
Decker, Walter E., A card system for keeping dental records and accounts, 19
Dental Board of New South Wales, 230
Dental Commissioners of Connecticut, 399, 479, 560
Dental education: a retrospective and prospective view, 600
Dentigerous cysts, 256
Development of hard tissue in the pulp of human teeth, 240
Device for extension crown, 401
Dissatisfied dental colleges, the, 629
DOMESTIC CORRESPONDENCE:
An apology to Dr. L. C. Bryan, of Basel, Switzerland, 153
Correction from Harvard, 225
Correction of article in March number, 314
Correction of stenographer's reports, 312
Dr. Black's correction, 641
Dr. Pierce on dental education and the St. Louis meeting, 736
First District Dental Society of New York, 395
National Association of Dental Faculties, 816
New Haven Dental Clinic, 539
"Occlusion" and "articulation," 900
Reply from "Trinity University," Toronto, 309
The present situation of the D.D.S., 897

Dr. Black's correction, 641
Dr. J. E. Garretson, dentist or surgeon? 949
Dr. Meyer L. Rhein's technique, 516
Dr. Ottolengui objects, 947
Dr. Peirce on dental education and the St. Louis meeting, 736

E

Eames, George F., M.D., The therapeutic use of the X-ray in the oral cavity, 356
The value of symmetry in education and character, 592
Earlier practice of dentistry in Hawaii, 267
EDITORIAL:
Antiseptic science in Chicago, 543
Correction, 735
Correction notice, 451
Correction of a serious error, 151
Dr. J. E. Garretson, dentist or surgeon? 949
Dr. Ottolengui objects, 947
Essentials in dental education, 942
Meeting of the "Faculties" at Washington, D. C., 547
Novel method in conferring degrees, 149
Reply to Harvard's correction, 218
The dissatisfied dental colleges, 629
The educational retrograde at St. Louis, 733
The Fourth International Dental Congress, 810
The golden anniversary banquet, 302
"The last call," 632
The National Association of Dental Faculties, 448
The reply of "Trinity University," 392
The retrograde fractional meeting at St. Louis, 634
The social standing of dentists, 887
Will gold-foil cease to be chief of filling-materials? 65
Will the dental colleges reverse their decision? 144
Essentials in dental education, 942
Evolution of standards in dental education, 698

F

"F. D. I." International Dental Federation, 142, 562, 661, 789
Fine, William Middleton, D.D.S., A method for reproducing the natural contour of artificial teeth on the lingual and palatal surfaces of artificial dentures, 569
First Dental Society of New York, 395
First District Dental Society of New York, 320
Fitzhardinge, H. C., D.D.S., Pyorrhœa alveolaris: a case in practice, 705
Fletcher, M. H., D.D.S., M.D., Pathological irregularities, 677
Florida State Dental Society, 87

Foreign Correspondence:
Dr. Bryan on prophylaxis and criticisms, 69
Form of agreement of the State Board of New Jersey, 86
Fourth International Dental Congress, 457, 462, 471, 554, 561, 579, 642, 648, 656, 657, 659, 797, 810, 820, 872
Franklin, Milton, M.D., Radiotherapy in some malignant conditions of the mouth, 905

G

Georgia State Dental Society, 480
Giblin, Thomas J., D.M.D., Teeth during pregnancy, 513
Gillett, Henry W., D.M.D., Device for extension crown, 401
Perplexities in connection with extraction of teeth, 368
Spalding's porcelain jacket crown, 362
Gilmer, Thomas L., M.D., D.D.S., Multiple fracture of the lower jaw complicated by double fracture of the upper jaw, 847
Golden anniversary banquet, 302
Grant, George F., D.M.D., A review of some methods of crowning teeth, 407
Great amalgam fallacy, the, 34
Green, Leo, A.B., D.M.D., A plea for thorough dental education, 585

H

Hales, Leonard C., D.D.S., An instructive case of hypertrophied pulp, 702
Hartford Dental Society, 958
Harvard Dental Alumni Association, 744
Hickman, Dr. H. B., Osteomyelitis: a case in practice, 111
High-fusing porcelain for dental purposes, the superiority of, 414
Hogue, T. Wilson, D.M.D., Root-filling, 704
Home manufacture of formaldehyde for sterilizing purposes, 364
Hypnotism, 686

I

Illinois State Dental Society, 319
Impacted teeth: their diagnosis, liberation, and extraction, 321
Institute of Dental Pedagogics, 957
International Dental Congress—report of committee on prize essays, 820
Interstate Dental Fraternity of the United States and Canada, 88, 663
Iowa State Dental Society, 230

J

Janlusz, Henry J., D.D.S., Suppurative cleft palates and devices for the same, 113

K

Kentucky State Dental Association, 318
Kirk, Edward C., D.D.S., Sc.D., The Fourth International Dental Congress, 579
Kyle, D. Braden, M.D., The relation of the chemistry of the saliva (sialosemeiology) and nasal secretions to diseases of the mucous membrane of the mouth and upper respiratory tract, 94

L

Latham, V. A., M.D., D.D.S., F.R.M.S., Neoplasm (epithelioma) of the pulp, 825

Lebanon Valley Dental Association, 567

M

Malignant growths in the mouth, 911

Marshall, John S., M.S., M.D., Dental education: a retrospective and prospective view, 600

Massachusetts Dental Society, 63

Mayer, Frank R., D.D.S., Pyorrhœa alveolaris, 195

McCullough, P. B., D.D.S., A suspension partial denture, 105

    Non-expanding plaster, 577

Meeting of the "Faculties" at Washington, D. C., 547

Method for reproducing the natural contour of artificial teeth on the lingual and palatal surfaces of artificial dentures, 569

Minnesota State Board of Dental Examiners, 229, 400

Minnesota State Dental Association, 228

MISCELLANY:

- A dangerous feature of arsenical paste, 157
- Adrenalin: a new hæmostatic, 227
- A modified needle for pressure anæsthesia, 742
- A more simple way of making a crown, 553
- Asbestos and alcohol as an investment, 157
- A useful pain obtundent, 158
- Excess of solder to be avoided in porcelain work, 159
- Grind lower teeth rather than force down, 226
- Keep a record of your treatments, 818
- Lead-pencil erasing points as dental tools, 158
- Local analgesia by adrenalin-eucaine, 396
- Method of tipping bridge teeth, 819
- New application of soft or velum rubber, 553
- Poisoning by corrosives, 740
- Reduction of tendency to hemorrhage, 227
- Repairing fractured casts, 817
- Retaining porcelain inlays, 157
- Temporary filling, 743
- The abuse of the gold cap, 226
- To prevent soreness of the lips after operating, 817
- To remove oxide from German silver, 227
- Treatment of contracture of joints with Röntgen rays, 819

Mississippi Valley Medical Association, 743

Moffatt, R. T., The art of carving and baking porcelain teeth and crowns, 1

Montana State Dental Society, 318

Morgenstern, Michael, Some histological facts that contradict the generally accepted odontoblast theory, 161

Multiple fracture of the lower jaw complicated by double fracture of the upper jaw, 847

### N

National Association of Dental Examiners, 229, 461, 561, 660, 821
National Association of Dental Faculties, 397, 448, 563, 816
Neoplasm (epithelioma) of the pulp, 825
New Haven Dental Association of Connecticut, 231
New Haven Dental Clinic, 639
New Jersey State Board of Registration and Examination in Dentistry, 318, 823
New Jersey State Dental Society, 229, 470
Non-expanding plaster, 577
Northern Indiana Dental Society, 399, 744, 823
Northern Iowa Dental Society, 568
Northern Ohio Dental Association, 480, 739
Novel method in conferring degrees, 149

### O

OBITUARY:
Ash, Mr. William, 84
Avery, Dr. Otis, 315
Resolutions of respect to, 456
Benton, Dr. J. H., 903
Chance, Dr. George H., 901
Odell, Dr. Francis M., resolutions of respect to, 85
Rust, Dr. Edgar Rose, 738
Stevens, Dr. Stephen G., 902
Taft, Dr. Jonathan, resolutions of respect to, 225, 739
Turner, James Smith, 551
Welch, Dr. T. B., 154
"Occlusion" and "articulation," 900
Ohio State Dental Society—thirty-ninth annual meeting, 959
ORIGINAL COMMUNICATIONS:
A card system for keeping dental records and accounts, 19
A method for reproducing the natural contour of artificial teeth on the lingual and palatal surfaces of artificial dentures, 569
An instructive case of hypertrophied pulp, 702
Ankylosis of the jaws, 764
A plea for thorough dental education, 585
Apparent trifles: their importance in dental practice, 283
A reply to Dr. Rhein's answer, 505
A report on orthodontia, 843
A review of some methods of crowning teeth, 407
A suspension partial denture, 105
Dental education: a retrospective and prospective view, 600
Dentigerous cysts, 256
Device for extension crown, 401
Dr. Meyer L. Rhein's technique, 516
Home manufacture of formaldehyde for sterilizing purposes, 364

Original Communications:
Hypnotism, 686
Impacted teeth: their diagnosis, liberation, and extraction, 321
Malignant growths in the mouth, 911
Multiple fracture of the lower jaw complicated by double fracture of the upper jaw, 847
Neoplasm (epithelioma) of the pulp, 825
Non-expanding plaster, 577
Orthodontic facial orthomorphia—importance of differentiation in cases of apparent mandibular protrusion, 193
Osteomyelitis: a case in practice, 111
Pathological irregularities, 677
Pericemental abscess, 916
Perplexities in connection with extraction of teeth, 368
Pulp degeneration, 745
Pyorrhœa alveolaris, 195, 274
Pyorrhœa alveolaris: a case in practice, 705
Radiotherapy in pyorrhœa alveolaris, and dental radiography, 495
Radiotherapy in some malignant conditions of the mouth, 905
Recent methods in the administration of anæsthetics, 665
Reply to Dr. E. K. Wedelstaedt, 183
Report of a case of Vincent's angina and stomatitis, 851
Report of the demonstrator of operative dentistry of the G. V. Black Dental Club (Inc.), of St. Paul, for the October, 1903, meeting, 23
Root-filling, 704
Some histological facts that contradict the generally accepted odontoblast theory, 161
Some methods of teaching elementary operative dentistry, 411
Some phases of dental education, 696
Some thoughts regarding methods, and a new appliance for moving dislocated teeth into position, 481
Spalding's porcelain jacket crown, 362
Suggestions relating to the examination of the teeth, 190
Suppurative cleft palates and devices for the same, 113
Teeth during pregnancy, 513
The advantages of co-operation in our profession, 43
The art of carving and baking porcelain teeth and crowns, 1
The collection of bills, 523
The development of hard tissue in the pulp of human teeth, 240
The diatoric tooth in bridge-work, 233
The earlier practice of dentistry in Hawaii, 267
The evolution of standards in dental education, 698
The Fourth International Dental Congress, 579
The great amalgam fallacy, 34
The interest of the dentist *versus* the interest of the public, 706.
The relation of the chemistry of the saliva (sialo-semeiology) and nasal secretions to diseases of the mucous membrane of the mouth and upper respiratory tract, 94

ORIGINAL COMMUNICATIONS:
The superiority of high-fusing porcelain for dental purposes, 414
The therapeutic use of the X-ray in the oral cavity, 356
The value of symmetry in education and character, 592
The vasomotor system of the pulp, 89
The vital action of the dental pulp, 759
Treatment of protruding and receding jaws by the use of the inter-maxillary elastics, 344
Orthodontic facial orthomorphia—importance of differentiation in cases of apparent mandibular protrusion, 193
Osteomyelitis: a case in practice, 111
Ottolengui, Rodriguez, The great amalgam fallacy, 34

P

Pathological irregularities, 677
Pennsylvania Association of Dental Surgeons, 232
Pennsylvania State Board of Dental Examiners, 397
Pennsylvania State Dental Society, 456
Pericemental abscess, 916
Perplexities in connection with extraction of teeth, 368
Pike, Charles G., D.M.D., Suggestions relating to the examination of the teeth, 190
Potter, William H., D.M.D., Some methods of teaching elementary operative dentistry, 411
Programme of the F. D. I., 663
Programme of the Fourth International Dental Congress, 648
Prophylaxis and Criticisms, Dr. Bryan on, 69
Pulp degeneration, 745
Pyorrhœa alveolaris, 195, 274
Pyorrhœa alveolaris: a case in practice, 705

R

Radiotherapy in pyorrhœa alveolaris, and dental radiography, 495
Radiotherapy in some malignant conditions of the mouth, 905
Reading Dental Society, 231
Recent methods in the administration of anæsthetics, 665
Relation of the chemistry of the saliva (sialo-semeiology) and nasal secretions to diseases of the mucous membrane of the mouth and upper respiratory tract, 94
Reply from "Trinity University," Toronto, 309
Reply of "Trinity University," 392
Reply to Dr. E. K. Wedelstaedt, 183
Reply to Harvard's correction, 218
Report of a case of Vincent's angina and stomatitis, 851
Report of the demonstrator of operative dentistry of the G. V. Black Dental Club (Inc.), of St. Paul, for the October, 1903, meeting, 23
Report on orthodontia, 843

Reports of Society Meetings:
Academy of Stomatology, 51, 137, 297, 443, 729, 783, 866
American Medical Association, Section on Stomatology, 200, 384, 398, 624, 772
" F. D. I." International Dental Federation, 142, 789
Fourth International Dental Congress, 797, 872
Massachusetts Dental Society, 63
The New York Institute of Stomatology, 45, 132, 372, 434, 536, 611, 719, 937
Retrograde fractional meeting at St. Louis, 634
Reviews of Dental Literature:
Bone-cysts, 926
Medical Education in the United States, 856
Oxygenated dentifrices, 527
Some notes on the enamel, 420
Some observations upon suppuration of the maxillary antrum, 119
Somnoforme, 291
Starting pits: their use and abuse, 431
The dangers of gelatin injections, 370
The mechanism of the eruption of the teeth, 530
The teaching of materia medica in medical schools, 862
The use of ethyl chloride as a general anæsthetic, 716
The uses of Swiss broaches, 533
Things of specialism and of this society that make for optimism, 712
Rhein, M. L., M.D., D.D.S., Reply to Dr. E. K. Wedelstaedt, 183
Root-filling, 704

S

Sanger, R. M., D.D.S., The diatoric tooth in bridge-work, 233
Smith, D. D., D.D.S., M.D., Pericemental abscess, 916
Smith, Dr. F. Milton, Apparent trifles: their importance in dental practice, 283
Social standing of dentists, 887
Some histological facts that contradict the generally accepted odontoblast theory, 161
Some methods of crowning teeth, a review of, 407
Some methods of teaching elementary operative dentistry, 411
Some phases of dental education, 696
Some thoughts regarding methods, and a new appliance for moving dislocated teeth into position, 481
South Dakota State Board of Dental Examiners, 397, 904
Southern Branch, National Dental Association, 160
Southern Dental Society of New Jersey, 231
Southwell, Dr. Charles, Dr. Myer L. Rhein's technique, 516
Southwestern Iowa Dental Society, 744
Spalding's porcelain jacket crown, 362
Stanley, Ned A., The advantages of co-operation in our profession, 43
Suggestions relating to the examination of the teeth, 190

Suppurative cleft palates and devices for the same, 113
Supreme Chapter, Delta Sigma Delta, 568, 662
Suspension partial denture, 105.

T

Talbot, Eugene S., M.S., D.D.S., M.D., LL.D., Pulp degeneration, 745
The vasomotor system of the pulp, 89
Teeth during pregnancy, 513
The diatoric tooth in bridge-work, 233
The educational retrograde at St. Louis, 733
The interest of the dentist *versus* the interest of the public, 706
" The last call," 632
The New York Institute of Stomatology, 45, 132, 159, 372, 434, 536, 611, 719, 937
The present situation of the D.D.S., 897
Therapeutic use of the X-ray in the oral cavity, 356
Thirty-sixth anniversary meeting of the First District Dental Society of the State of New York, 958
Thorough dental education, a plea for, 585
Tousey, Sinclair, A.M., M.D., Radiotherapy in pyorrhœa alveolaris, and Dental Radiography, 495
Treatment of protruding and receding jaws by the use of the intermaxillary elastics, 344

V

Value of symmetry in education and character, 592
Vasomotor system of the pulp, the, 89
Vital action of the dental pulp, 759

W

Walker, William Ernest, D.D.S., M.D., Orthodontic facial orthomorphia—importance of differentiation in cases of apparent mandibular protrusion, 193
Wedelstaedt, E. K., A reply to Dr. Rhein's answer, 505
Report of the demonstrator of operative dentistry of the G. V. Black Dental Club (Inc.), of St. Paul, for the October, 1903, meeting, 23
Wheeler, Dr. Herbert L., Home manufacture of formaldehyde for sterilizing purposes, 364
The superiority of high-fusing porcelain for dental purposes, 414
Whitney, Dr. J. M., The earlier practice of dentistry in Hawaii, 267
Will gold-foil cease to be chief of filling-materials? 65
Will the dental colleges reverse their decision? 144
Wisconsin State Dental Society, 568

